# Hodgkin lymphoma detection and survival: findings from the Haematological Malignancy Research Network

**DOI:** 10.3399/bjgpopen19X101668

**Published:** 2019-12-11

**Authors:** Maxine JE Lamb, Eve Roman, Debra A Howell, Eleanor Kane, Timothy Bagguley, Cathy Burton, Russell Patmore, Alexandra G Smith

**Affiliations:** 1 Research Fellow, Epidemiology & Cancer Statistics Group (ECSG), Department of Health Sciences, University of York, York, UK; 2 Professor, Epidemiology & Cancer Statistics Group (ECSG), Department of Health Sciences, University of York, York, UK; 3 Senior Research Fellow, Epidemiology & Cancer Statistics Group (ECSG), Department of Health Sciences, University of York, York, UK; 4 Research Fellow, Epidemiology & Cancer Statistics Group (ECSG), Department of Health Sciences, University of York, York, UK; 5 Data Analyst, Epidemiology & Cancer Statistics Group (ECSG), Department of Health Sciences, University of York, York, UK; 6 Consultant Haematologist, Haematological Malignancy Diagnostic Service, St James’s University Hospital, Leeds, UK; 7 Consultant Haematologist, Queen’s Centre for Oncology and Haematology, Hull and East Yorkshire Hospitals, Cottingham, UK; 8 Reader, Epidemiology & Cancer Statistics Group (ECSG), Department of Health Sciences, University of York, York, UK

**Keywords:** cancer staging, data collection, delayed diagnosis, prognosis, real-world data, routes to diagnosis

## Abstract

**Background:**

Hodgkin lymphoma is usually detected in primary care with early signs and symptoms, and is highly treatable with standardised chemotherapy. However, late presentation is associated with poorer outcomes.

**Aim:**

To investigate the relationship between markers of advanced disease, emergency admission, and survival following a diagnosis of classical Hodgkin lymphoma (CHL).

**Design & setting:**

The study was set within a sociodemographically representative UK population-based patient cohort of ~4 million, within which all patients were tracked through their care pathways, and linked to national data obtained from Hospital Episode Statistics (HES) and deaths.

**Method:**

All 971 patients with CHL newly diagnosed between 1 September 2004–31 August 2015 were followed until 18th December 2018.

**Results:**

The median diagnostic age was 41.5 years (range 0–96 years), 55.2% of the patients were male, 31.2% had stage IV disease, 43.0% had a moderate–high or high risk prognostic score, and 18.7% were admitted via the emergency route prior to diagnosis. The relationship between age and emergency admission was U-shaped: more likely in patients aged <25 years and ≥70 years. Compared to patients admitted via other routes, those presenting as an emergency had more advanced disease and poorer 3-year survival (relative survival 68.4% [95% confidence interval {CI} = 60.3 to 75.2] versus 89.8% [95% CI = 87.0 to 92.0], respectively [*P*<0.01]). However, after adjusting for clinically important prognostic factors, no difference in survival remained.

**Conclusion:**

These findings suggest that CHL survival as a whole could be increased by around 4% if the cancer in patients who presented as an emergency had been detected at the same point as in other patients.

## How this fits in

In order to improve survival, the *NHS Long Term Plan* aims to increase the number of cancers diagnosed at stage I–II from 50% to 75%.

In CHL, a highly treatable haematological malignancy, emergency admission prior to diagnosis was used as a proxy for delayed diagnosis. It was found that younger and older patients, patients with comorbidities, and those with advanced disease were more likely to experience delay.

As expected, survival was poorer in this group, but importantly, after adjusting for prognostic factors, no differences in outcome were seen; indicating that if the cancer in patients presenting via an emergency route was diagnosed at the same point as patients presenting via other routes, outcomes would be equal.

These findings support the new NHS initiative, but it remains to be seen whether the targets are achievable in cancers with symptoms that are often vague, intermittent, and slow to progress.

## Introduction

The relationship between late diagnosis and poor outcome is recognised for many cancers, with delay often resulting in more advanced disease, worse survival, increased risk of complications, and impaired quality of life.^1–3^ Hence, over recent decades the UK Department of Health has introduced a series of interventions aimed at facilitating earlier diagnosis. In primary care settings, this includes referral guidance to aid GP identification of cancer symptoms and the introduction of suspected cancer pathways to minimise time to specialist secondary care consultation.^4^ The latter includes ‘urgent’ referral pathways (consultation with a hospital specialist within 2 weeks, known as the ‘2-week wait’) and ‘very urgent’ (within 48 hours) for children and young adults.^4^ More recently, the *NHS Long Term Plan* ^5^ introduced further important targets aimed at improving cancer outcomes, including an increase in the proportion of cancers identified at an early stage, from around one-half at present to three-quarters by 2028.

Hodgkin lymphoma has two distinct subtypes: CHL and nodular lymphocyte predominant Hodgkin lymphoma (NLPHL). The present report is restricted to CHL, which accounts for around 90% of all newly diagnosed Hodgkin lymphomas and has a characteristic bimodal age distribution with a peak in younger adults and a further peak in older adults.^6^ CHL can be further subdivided into nodular sclerosing, mixed cellularity, lymphocyte rich, and lymphocyte depleted Hodgkin lymphoma. Timely diagnosis is of particular interest in the context of CHL, since it typically presents with early signs and symptoms and is highly treatable with standardised chemotherapy; the 5-year survival is around 85%,^7–9^ decreasing to around 70% in those diagnosed at an advanced stage.^7^ By contrast, the clinical course of the rarer NLPHL is generally indolent, immediate treatment is not always required, and relative survival approaches that of the general population.^9–12^

CHL presents several challenges to detection in primary care: it is comparatively rare, accounting for <1% of all cancers diagnosed each year in the UK^8^ and can be diagnosed at any age.^7^ Furthermore, while patients may present with classic symptoms including neck lumps, itching, and B symptoms (such as night sweats, fever, and weight loss), these, along with other symptoms, can be intermittent and slow to progress, as well as being indicative of more commonly occurring conditions.^9,13,14^

However, information on the relationship between presentation mode, disease stage, and survival for CHL is lacking. With the aim of providing contemporary population-based evidence to address this gap, clinical data and HES from an established UK patient cohort^15^ were used to explore the relationship between markers of diagnostic delay and survival in patients with newly diagnosed CHL.

## Method

Data are from the UK’s Haematological Malignancy Research Network (HMRN; www.hmrn.org) which, with a catchment population of around 4 million that is sociodemographically representative of the UK as a whole, generates ‘real-world’ data that can be extrapolated to the general patient population (adults and children). Full details of HMRN’s methods and ethical approvals have been published elsewhere.^15,16^ Briefly, initiated in September 2004, HMRN operates on a legal basis that permits all diagnostic, prognostic, treatment, and outcome data to be collected from clinical records, as well as linkage to nationwide information on deaths, cancer registrations, and HES without explicit consent. All diagnoses across HMRN’s 14 hospitals (~2400 per year) are made and coded to the latest World Health Organization International Classification of Diseases for Oncology (WHO ICD-O3) by specialist haematopathologists at a single fully-integrated laboratory, the Haematological Malignancy Diagnostic Service (www.hmds.info).

The present report includes data on 971 patients newly diagnosed with CHL between 1 September 2004 and 31 August 2015, all of whom were followed-up until 18 December 2018. Disease stage was based on the modified Ann Arbor classification (I-IV),^17^ performance status was assessed using the Eastern Oncology Cooperative Group’s (ECOG) scale (ranging from 0 ‘able to carry out normal activity without restriction’ to 4 ‘completely disabled; cannot carry out any self-care’),^18^ and the Hodgkin lymphoma-specific International Prognostic Score ([IPS] Hasenclever Index, developed for use in patients with advanced disease) was calculated for all patients.^19^ Patients were classified as treated with curative intent if they received intensive chemotherapy, as per national guidelines.^6^ Presence or absence of disease-associated systemic symptoms (B symptoms) was also recorded. Additional information on preceding comorbidities was obtained from inpatient HES using validated ICD-10 codes for the 17 conditions in the Charlson Comorbidity Index.^20–23^ Pre-diagnostic emergency admissions were identified in HES using ‘Routes-to-Diagnosis’ methods, which include: an emergency inpatient admission originating via Accident & Emergency attendance, GP, Bed Bureau transfer, or consultant outpatient clinic within 28 days of diagnosis.^24–26^ The income domain of the Index of Multiple Deprivation (IMD), grouped into quintiles (with Q1 representing the most affluent fifth of the population), was used as a marker of socioeconomic status.^27^

### Statistical analysis

Analyses were conducted using Stata (version 15.1) and R (version 3.2.2). Logistic regression was used to calculate odds ratios (ORs). Cox proportional hazards regression and Stata’s ‘strel’ program, which is based on the maximum likelihood method for individual records,^28^ were used to calculate overall survival and relative survival, respectively. The latter is a standard approach commonly used in population-based studies using national life tables^29^ to take into account other causes of death. Adjusted survival curves were produced using the average approach. Firstly, propensity scores were calculated, based on the probability of emergency admission predicted using logistic regression adjusted for ECOG status, B symptoms, disease stage, and other specific components of the IPS (such as albumin, haemoglobin, and lymphocyte count). These probability predictions were then scaled to the proportion of patients in each group to give the adjusted curve weights. Cox proportional hazards regression survival curves were then estimated for all combinations of the covariates included in the propensity scores plus age and curative treatment, and a weighted mean of the curves was calculated to adjust for the mix among patients, with emergency admission compared to all other routes.^30^

## Results

**Figure 1.**
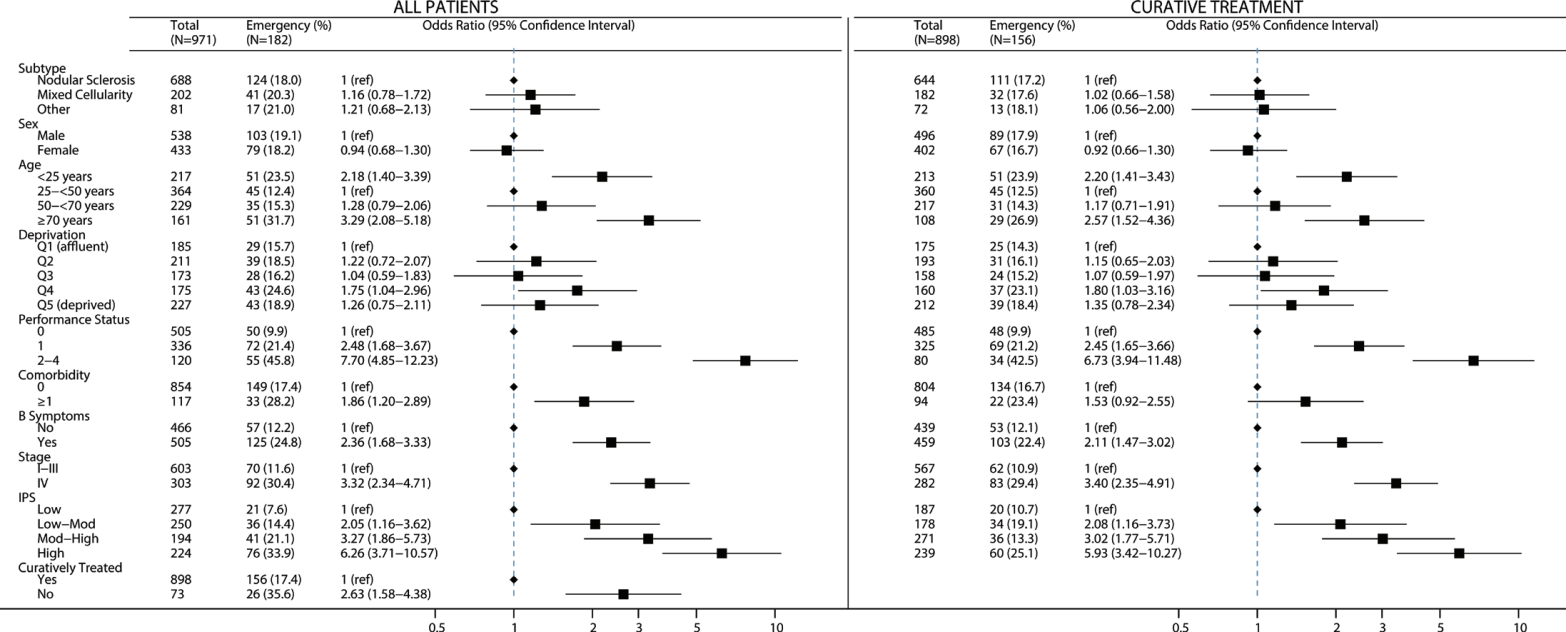
Patients with classical Hodgkin lymphoma distributed by demographic and clinical prognostic characteristics, with ORs (95% CIs) for diagnosis following emergency admission presented for all patients and those treated with curative intent (data from HMRN diagnoses 2004–2015). IPS = International Prognostic Score

The median diagnostic age of the 971 patients with CHL was 41.5 years and 55.2% were male. Nodular sclerosis was the most common subtype (*n* = 688, 70.9%, median age 36.0 years), followed by mixed cellularity (*n* = 202, 20.8%, 59.6 years), and lymphocyte rich (*n* = 26, 2.7%, 51.6 years). CHL subtype was not characterised for the remaining patients (*n* = 55, 5.7%, 53.6 years). In total, 182 (18.7%) were diagnosed following emergency admission; 303 patients (31.2%) had stage IV disease at diagnosis; 418 (43.0%) had a moderate–high or high-risk IPS; and 898 (92.5%) were treated with curative intent (Figure 1).

The relationship between emergency admission and age was U-shaped, with patients aged <25  years or ≥70 years the most likely to present via this route (Figure 1, Supplementary Table 1): the respective ORs were 2.18 (95% CI = 1.40 to 3.39) and 3.29 (95% CI = 2.08 to 5.18). Patients with an emergency admission tended to have higher stage disease (OR 3.32, 95% CI = 2.34 to 4.71); more comorbidities (OR 1.86; 95% CI = 1.20 to 2.89); at least one B symptom (OR 2.36, 95 % CI = 1.68 to 3.33); and poorer performance status (OR 7.70, 95% CI = 4.85 to 12.23). No differences were detected by CHL subtype, sex, or socioeconomic status. The 73 patients who were not treated with curative intent were also more likely to have had an emergency admission (OR 2.63, 95% CI = 1.58 to 4.38); but their exclusion had no marked effect on the relationship between emergency admission and patient demographic and prognostic characteristics (Figure 1).

**Figure 2.**
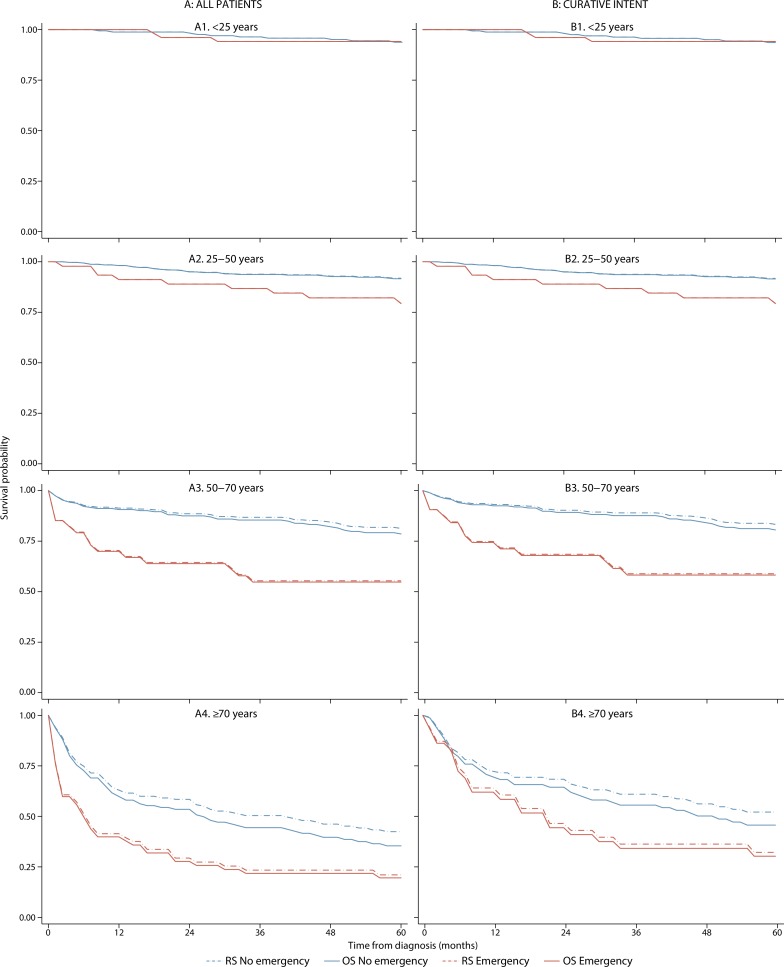
Classical Hodgkin lymphoma overall survival (OS) and relative survival (RS) curves for all patients (A) and those treated with curative intent (B), stratified by emergency admission status and age (data from Haematological Malignancy Research Network diagnoses 2004–2015)

Three-year relative survival estimates for all patients and those treated curatively were 86.7% (95% CI = 84.0 to 89.0) and 90.1% (95% CI = 87.5 to 92.1), respectively (Supplementary Table 2). As expected, given the strength of the associations seen in Figure 1, outcomes were significantly poorer for patients with an emergency admission compared to those diagnosed via other routes: the 3-year relative survival estimates were 68.4% (95% CI = 60.3 to 75.2) and 89.8% (95% CI = 87.0 to 92.0), respectively. Figure 2 shows overall and relative survival for emergency compared to non-emergency admissions by age strata, for all patients (A1–4) and those treated with curative intent (B1–4). In patients aged ≤25 years (Figure 2: A1, B1), no survival differences are evident between those with and without emergency admission. However, at older ages diagnosis following an emergency admission was associated with significantly poorer survival than diagnosis following other routes: respective 3-year relative survival estimates for emergency and non-emergency admissions were 55.4% (95% CI = 36.8 to 70.5) and 86.6% (95% CI = 80.8 to 90.1) in patients 50–70 years, and 23.4% (95% CI = 12.4 to 36.3) and 50.4% (95% CI =39.5 to 60.3) in patients ≥70 years of age (Figure 2: A3, A4).

**Figure 3.**
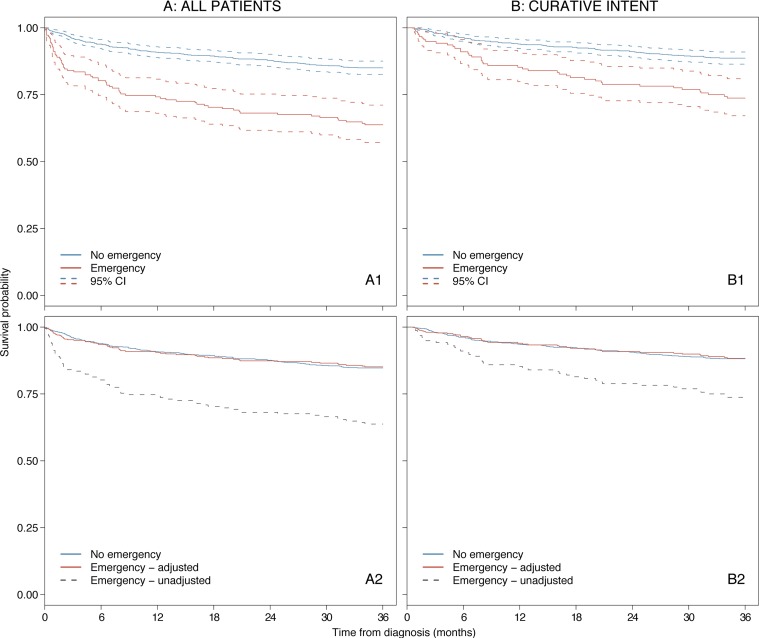
Classical Hodgkin lymphoma survival curves for all patients (A) and those treated with curative intent (B). Overall survival in non-emergency versus emergency admission with 95% CIs (A1, B1) and non-emergency versus emergency adjusted for prognostic factors (A2, B2).^a^ (data from HMRN diagnoses 2004–2015) ^a^Survival adjusted for age, performance status (ECOG), B symptoms (yes/no), disease stage, specific components of IPS (albumin, haemoglobin, and lymphocyte count), and treatment with curative intent (yes/no) (A2 only). Survival curves are weighted so that the patient mix with respect to ECOG status, B symptoms, disease stage, and specific components of IPS among those presenting as an emergency is similar to those who presented via other routes. ECOG = Eastern Oncology Cooperative Group. IPS = International Prognostic Score

For emergency admissions, mortality was particularly high immediately after diagnosis (Figure 3: A1), although the disparity was marginally less marked when analyses were restricted to patients treated with curative intent (Figure 3: B1). However, for patients with an emergency admission surviving 1-year (conditional survival) mortality did not return to the background rate, with a 3-year relative survival of 88.7% (95% CI = 81.0 to 93.3) compared to 96.1% (95% CI = 94.0 to 97.4) in those without an emergency admission. Overall survival estimates at 1, 3, and 5 years, stratified by prognostic factors and emergency admission, are included in Supplementary Table 3. Importantly, when the prognostic profile of patients with emergency admission was adjusted to mirror that of other routes, the differences between groups disappeared (Figure 3: A2 and B2). The factor most influential in these adjustments was performance status, while albumin and haemoglobin also had an impact (Supplementary Figure 1). In practice, this implies that if patients who presented by the emergency route were diagnosed at the same point in their disease course as those who did not, the 3-year overall survival in the whole study population would equal that in the group who did not have an emergency admission; increasing from 81.1% (95% CI = 78.5 to 83.5) to 85.2% (95% CI = 82.5 to 87.5); a difference of around 4% (Supplementary Table 3).

## Discussion

### Summary

Linking contemporary high-quality clinical information with data contained in NHS administrative databases, this is the first population-based study to examine CHL-specific evidence about the impact of emergency admission and disease stage on survival. This study clearly identifies the substantial benefits to be gained from early CHL diagnosis, principally among older adults. Within this large UK population-based cohort (diagnoses 2004–2015, followed-up to December 2018), almost one in five patients were diagnosed following an emergency hospital admission, and these were more likely be diagnosed with stage IV disease. This route to diagnosis was associated with significantly worse survival than all other modes of presentation, with prognostic data indicating that the difference was largely driven by more advanced disease. Indeed, the striking disparity, seen for all patients and those treated with curative intent, disappeared when standard prognostic factors were adjusted for; raising the possibility that CHL survival as a whole could be increased by around 4% if the cancer in patients who presented as an emergency had been detected at the same point as in other patients. A potentially modifiable difference of this magnitude is broadly similar to that seen in successful treatment trials.^31,32^

### Strengths and limitations

This study’s findings can be extrapolated to the national population because the sociodemographic profile of HMRN’s catchment population, which at around 4 million accounts for 6% of the UK’s estimated total, is broadly representative of the UK as a whole and clinical practice adheres to national guidelines.^15^ Critically, the fact that diagnoses within HMRN are made and coded by clinical experts, also enabled the distinction between CHL and NLPHL, which are often combined in population-based studies despite substantial differences in presentation, management, and outcome.^10,11,33^ Moreover, in addition to this, it was also possible to differentiate between CHL subtypes; patients with mixed cellularity CHL had poorer 3-year relative survival than those with nodular sclerosing CHL: 79.5% (95% CI = 72.0 to 85.1) and 89.4% (95% CI = 86.4 to 91.8) respectively (Supplementary Table 2), and the impact of emergency admission on relative survival was greatest in patients with mixed cellularity CHL (37% difference versus 15% difference: Supplementary Table 2). However, as mixed cellularity CHL is more common in older patients and nodular sclerosing CHL in younger patients (median age 59.6 years and 36.0 years respectively), the difference in outcomes is likely due, at least in part, to age. Importantly, in contrast to clinical trials, selection bias is not an issue within HMRN as all diagnoses are automatically included; and the availability of detailed clinical data enables potentially confounding prognostic factors to be considered in the analyses.

With respect to the use of HES, the authors previously demonstrated an association between emergency admission and survival in aggressive non-Hodgkin lymphoma that, in contrast to the findings for CHL presented here, remained after adjusting for prognostic and demographic factors.^34^ Undoubtedly, however, lack of primary care data limits the analysis that can be performed. A study by Abel and colleagues reported that 11.9% of patients with Hodgkin lymphoma (all subtypes combined) who presented via an emergency route and participated in the 2010 English Cancer Patient Experience Survey, had not visited their GP prior to admission.^35^ Unfortunately, it was not possible to investigate patterns and referrals from primary care, as national linkage to these data is currently not possible. Furthermore, as Hodgkin lymphoma is a rare disease and despite the inclusion of 971 cases in this population-based study, after stratification by emergency admission the number of subjects in some categories is small, making comparisons difficult.

### Comparison with existing literature

This study’s findings are broadly similar to those published by the National Cancer Registration and Service (NCRAS) in terms of overall survival for all Hodgkin lymphoma subtypes combined, and emergency admission frequency.^36^ Relative survival estimates are also comparable to those published by the US’s Surveillance, Epidemiology and End Results programme.^37^ However, the general absence of clinical data on prognostic factors and treatment, mean that more granular comparisons in these registries are not possible. However, analyses of NCRAS data for several common cancers (colorectal, cervical, breast, lung, and prostate) reported that older age and advanced stage disease at diagnosis was predictive of emergency admission.^38^ In these cancers, after adjustment for age, stage, and comorbidity, emergency admission was still associated with poorer 1-year survival.^38^ However, they could not adjust for performance status, which in this study’s data has the strongest influence on survival.

The relationship between age at diagnosis and odds of having an emergency admission prior to diagnosis was U-shaped in the current data. The higher proportion of emergency admissions in younger patients may be a result of the 48-hour referral recommended in children and young adults suspected to have cancer,^4^ and the increased presence of comorbidities in older patients may impact on the higher odds of emergency admission.^39^ Importantly, no difference in outcomes among younger (<25 years) patients with and without an emergency admission was seen. This was in contrast to older adults (≥70 years) whose survival was poorer if diagnosed following an emergency admission, an association that remained in those treated curatively. However, it is well recognised that despite CHL being curable, outcomes in older patients are poor, which is generally attributed to lower tolerance of standardised chemotherapy regimens, increased comorbidities, and poorer performance status.^6,40,41^

### Implications for research and practice

The vast majority of patients seek GP help for their symptoms at some time prior to cancer identification, including those who later go on to have a pre-diagnostic emergency admission.^35,42^ The opportunity to identify potential symptoms and refer patients earlier for specialist hospital consultation cannot therefore be ignored. Presently, however, raising suspicion of CHL, and indeed other haematological malignancies in primary care, is considered difficult,^43,44^ not least because of the absence of clear symptoms, specific diagnostic blood tests, and realistic screening programmes, which mean repeat appraisal, help-seeking, and hospital referral is often required prior to diagnosis.^13,45^ Analyses of time trends of English data have shown an overall decrease in diagnoses following emergency presentation of 3% over the period 2006–2013; however, the reduction seen for Hodgkin lymphoma was only 1%.^46^ Although the means of resolving such issues are far from obvious, research to further understanding of patient–healthcare professional interactions would inform the development of initiatives to promote early diagnosis.

This study’s findings demonstrate the importance of existing national strategies including: safety netting in primary care, with timely post-investigation review as well as scheduled and/or patient-initiated follow-up; Rapid Diagnostic Centres for use when presenting symptoms are vague and do not meet urgent referral criteria; and faster diagnosis standard.^4,5^ Specific to Hodgkin lymphoma, urgent referral and investigation of older patients has been suggested for unexplained lymphadenopathy.^14^ The effectiveness of such measures in increasing the proportion of early stage cancer diagnoses, as described in the *NHS Long Term Plan*,^5^ remains to be seen.

In conclusion, one-fifth of patients were diagnosed with CHL following an emergency admission. These patients had more advanced disease and had poorer survival. As the difference in survival did not remain after adjustment for prognostic factors, the findings suggest that if CHL had been detected at the same point as in patients presenting via other routes, the 3-year survival from CHL in the whole study population could be increased by approximately 4%.
